# Are the effects of stress on antenatal depression mediated by self-esteem and moderated by social support?: a cross-sectional study

**DOI:** 10.4069/whn.2024.10.18.1

**Published:** 2024-12-30

**Authors:** Eunjoo Lee

**Affiliations:** Department of Nursing, Kyungnam University, Changwon, Korea

**Keywords:** Depression, Pregnancy, Self concept, Social support, Stress

## Abstract

**Purpose:**

Social support and self-esteem play crucial roles in influencing the mental health of pregnant women. This study explored the moderating role of social support and the mediating role of self-esteem in the relationship between pregnancy-related stress and antenatal depression among pregnant women.

**Methods:**

This cross-sectional study included 180 pregnant women who visited the obstetrics outpatient clinic at a hospital in Changwon, Korea, between November 22 and December 30, 2022. Data were collected through structured questionnaires that assessed antenatal depression, pregnancy stress, self-esteem, and social support. The data were analyzed using descriptive statistics, Pearson correlation coefficients, and PROCESS Macro model 5.

**Results:**

The mean gestational age of the participants was 23 weeks. The mean score for pregnancy stress was 63.51±14.33, indicating a moderate level of self-esteem and social support were high, with scores of 31.06±4.83 and 41.96±7.73, respectively. Prenatal depression was notably low, averaging 6.51±4.59. It showed a significant positive correlation with pregnancy stress (r=.52, *p*<.001) and negative correlations with self-esteem (r=–.49, *p*<.001) and social support (r=–.24, *p*=.001). Self-esteem acted as a mediator in the relationship between pregnancy stress and antepartum depression among pregnant women, with an indirect effect of .05 and a 95% confidence interval of .02 to .08. Additionally, social support moderated the impact of pregnancy stress on antepartum depression (B=–.01, *p*=.036).

**Conclusion:**

The study emphasizes the need for a comprehensive approach to maternal mental health during pregnancy, which should include stress management, self-esteem enhancement, and social support interventions. Addressing these interconnected factors is crucial for promoting maternal well-being and reducing the incidence of prenatal depression.

## Introduction

Pregnancy is a critical time characterized by significant changes and adaptations in a woman’s life. During this period, women face an increased risk of mental health issues [1]. Research indicates that about 20% of pregnant women suffer from antenatal depression [2], which in severe cases, may necessitate medical intervention [1]. Symptoms of antenatal depression extend beyond sadness to include a loss of interest, diminished energy, impaired concentration, feelings of guilt, sleep disturbances, and reduced appetite [3]. Even mild symptoms of depression can adversely affect pregnancy and childbirth, potentially leading to issues such as preterm birth, low birth weight, and fetal growth restriction [4]. These conditions can also result in difficult temperament and behavioral regulation disorders in the child [5].

Antenatal depression arises from a mix of environmental, biological, psychosocial factors, and hormonal changes [1,6]. Risk factors for antenatal depression include pregnancy stress, maternal age, fear of childbirth, low socioeconomic status, being unmarried, previous pregnancy termination, smoking, gestational diabetes [7], preeclampsia, and complications such as premature rupture of membranes [4]. Among these, pregnancy stress is a primary risk factor for antenatal depression [8]. Recent studies have indicated that the risk of antenatal depression escalates with increased levels of pregnancy stress [9]. Specifically, the likelihood of experiencing depression during pregnancy rises by 1.32 times for each one-point increase in the recognized stress score [10]. Stress during pregnancy, stemming from emotional and physical changes, financial difficulties, and a lack of social support, further heightens the risk of antenatal depression [11]. However, the relationship between stress and depression is complex, involving a variety of contributing factors.

Among the various factors that influence antenatal depression, self-esteem plays a critical role [10]. Self-esteem is the individual’s assessment of their own worth, viewed as either positive or negative [12]. It significantly predicts success and happiness across different life domains, including relationships, work, and health [13]. Individuals with high self-esteem generally experience greater life satisfaction, fewer relationship issues, and are less prone to psychological problems [14]. Conversely, those with low self-esteem are more likely to exhibit symptoms of depression as they negatively react to adverse events [15]. Choi and Lee [16] describe a relationship where stress influences depression by first lowering self-esteem, which then heightens depression levels. This suggests that stressful events can indirectly affect depression through self-esteem [17], in addition to their direct impact [13]. The mediation pathway involving self-esteem indicates that stressful events might lead to depression, particularly if they result in lowered self-esteem [16]. Specifically, the physical and mental stresses associated with pregnancy can diminish a woman’s self-esteem, thereby increasing her susceptibility to depression [8,16]. Thus, demonstrating the mediating role of self-esteem in the relationship between pregnancy-related stress and antenatal depression could be pivotal in developing effective prevention strategies for this condition.

Social support plays a crucial role in preventing depression by helping pregnant women maintain a positive mindset [11]. Research from a meta-analysis indicates that the incidence of antenatal depression is 1.18 times higher among pregnant women with lower levels of social support, demonstrating a strong correlation between reduced social support and increased antenatal depression [18]. Furthermore, social support is widely recognized for its critical role in moderating the relationship between stress and psychological outcomes [19]. By buffering the effects of stress on depression, social support can make stress seem less severe than it actually is and enhance the ability to cope with stress [20]. Pregnancy is a time of significant physical and mental changes. Insufficient social support during this period can further increase the risk of antenatal depression due to stress [8,18,21,22]. These findings indicate that social support serves as a mediator between pregnancy-related stress and antenatal depression and that the influence of stress on antenatal depression can vary based on the level of social support available. Therefore, it is essential to conduct detailed research on the types of moderating effects that social support has on the relationship between pregnancy stress and antenatal depression.

Although previous studies conducted overseas [15-17,18,21-23] have focused on the role of social support and self-esteem in the relationship between pregnancy stress and antenatal depression, no research has analyzed the indirect relationship between self-esteem and social support simultaneously. In Korea, research has been conducted on the influencing factors of antenatal depression [10] and experiences of antenatal depression [11]. These studies primarily analyzed the direct relationship between antenatal depression and related variables, with limited exploration of the indirect pathways and the effects of interactions between these variables.

Therefore, this study explored the relationship between pregnancy stress, antenatal depression, self-esteem, and social support. It also seeks to assess the mediating role of self-esteem and the moderating role of social support in the relationship between pregnancy stress and antenatal depression. The ultimate goal is to contribute to the prevention of antenatal depression. The hypotheses of this study are outlined below.

Hypothesis 1. Pregnancy stress positively impacts antenatal depression.

Hypothesis 2. Self-esteem mediates the relationship between pregnancy stress and antenatal stress.

Hypothesis 3. Social support mediates the relationship between pregnancy stress and antenatal stress.

## Methods

Ethics statement: This study was approved by the Institutional Review Board of Kyungnam University (No. 1040460-A-2022-032). Informed consent was obtained from the participants.

### Study design

This descriptive correlational study aimed to explore the impact of pregnancy stress on antenatal depression, as well as to examine the mediating role of self-esteem and the moderating role of social support in the relationship between pregnancy stress and antenatal depression. The study adhered to the STROBE reporting guidelines (https://www.strobe-statement.org/).

### Participants

In total, 180 pregnant women who visited the outpatient department of obstetrics and gynecology at Hanmaeum Medical Center in Changwon, Korea were recruited through convenience sampling as participants in this study. The detailed inclusion criteria were as follows: (1) individuals aged 20 years and above, (2) those who were 4 to 42 weeks pregnant, and (3) those who understood the purpose of the study and voluntarily provided their consent to participate. The exclusion criteria include individuals receiving treatment for pregnancy complications, such as miscarriage, extrauterine gestation, and vaginal hemorrhage, among others.

Since the gestational sac can be observed from around 4 to 5 weeks of pregnancy, the study period was defined as 4 to 42 weeks, which encompasses full-term pregnancy. The sample size was calculated using G*Power 3.1.2 with the following parameters necessary for multiple regression analysis: an F-test, a significance level of .05, a test power of .90, a medium effect size of .15 [24], and 15 predictor variables (twelve general characteristics and three continuous variables). The minimum required sample size was determined to be 171. To account for a potential dropout rate of 10%, 190 participants were initially recruited. Ultimately, 180 surveys were included in the final analysis after excluding 10 that were improperly completed.

### Measurements

#### Antenatal depression

The Edinburgh Postnatal Depression Scale (EPDS), originally developed by Cox et al. [25], was adapted into Korean by Kim [26]. This version of the EPDS is employed to assess antenatal depression, despite its initial design for postnatal depression. Its applicability and validity for antenatal use are well-established [27]. The scale includes 10 items that evaluate symptoms experienced over the past week, such as depression, anxiety, nervousness, fear, guilt, and thoughts of self-harm. Responses are rated on a 4-point Likert scale, ranging from 0 to 3, with total scores varying from 0 to 30. In this study, which included all pregnant women irrespective of their depression risk, no cutoff point was applied. All items, except for 1, 2, and 4, were reverse-scored, meaning that a higher total score indicates greater levels of antenatal depression. At the time of its development [25], the tool’s reliability, measured by Cronbach’s α, was .87; in this study, it was .85.

#### Pregnancy stress

The pregnancy stress scale developed by Ahn [28] was utilized to assess the level of stress during pregnancy, and permission was granted for the use of this tool. This scale measures the stress experienced by pregnant women due to physiological, psychological, and physical changes. It comprises 26 items, divided into three categories: nine items focus on fetus-related stress, 11 items address stress concerning the pregnant women themselves, and six items pertain to spouse-related stress. Each item is rated on a 5-point Likert scale, ranging from 1 point (“not worried at all”) to 5 points (“always worried”), with total scores varying from 26 to 130. A higher score indicates greater pregnancy stress. At the time of its development [28], the tool’s reliability, as measured by Cronbach’s α, was .84, while in this study, it was .91.

#### Self-esteem

Self-esteem was assessed using Jeon’s [29] translated version of the Rosenberg Self-Esteem Scale, originally developed by Rosenberg [12]. This instrument consists of 10 items, divided equally between positive and negative self-esteem. Each item is rated on a 4-point Likert scale, ranging from 1 (“seldom”) to 4 (“always”), with total scores varying from 10 to 40. Items 1, 2, 3, 6, and 7 are scored directly, while the remaining items are reverse-scored. A higher total score indicates greater self-esteem. In Jeon’s [29] research, Cronbach’s α for reliability was .92, whereas in this study, it was .87.

#### Social support

The Multidimensional Scale of Perceived Social Support, developed by Zimet et al. [30] and translated by Shin and Lee [31], was utilized to assess social support. Permission was granted for the use of this instrument. It gathers data on perceived support from family, friends, and significant others. The scale consists of 12 items, divided equally into four items each addressing support from family, friends, and significant others. Each item is rated on a 5-point Likert scale, ranging from 1 (“strongly disagree”) to 5 (“strongly agree”), with the total score varying from 12 to 60. A higher score indicates greater perceived social support. In the study by Shin and Lee [31], Cronbach’s α was reported as .89, which matched the Cronbach’s α value found in this study.

#### General characteristics

The general characteristics recorded included age, marital status, employment status, monthly household income, marital satisfaction, weeks of pregnancy, whether the pregnancy was planned, number of pregnancies, presence of current children, pregnancy-related issues, personal history of depression diagnosis and treatment, and family history of depression.

### Data collection

Data were collected using structured questionnaires from November 22 to December 30, 2022. Prior to data collection, authorization was secured from the nursing department at H Hospital. Additionally, a notice for participant recruitment was displayed in the obstetrics and gynecology outpatient department of the hospital. The principal investigator discussed the study’s purpose and methodology with the chief nurse and the information desk staff in the obstetrics and gynecology outpatient department, seeking their cooperation in distributing the questionnaires. Individuals interested in participating, having seen the recruitment notice, were instructed to obtain the questionnaire from the information desk staff, complete it, and return it upon completion. The principal investigator provided a detailed explanation of the study’s purpose, procedures, data confidentiality, protection of personal information, potential benefits, possible discomforts, and the participants’ right to withdraw consent at any time. Following this, written consent was obtained from each participant. As a gesture of appreciation, participants received a preselected gift.

### Data analysis

Data were analyzed using IBM SPSS Statistics for Windows, version 23.0 (IBM Corp., Armonk, NY, USA). We calculated frequencies, percentages, means, and standard deviations to describe participants’ general characteristics, antenatal depression-related characteristics, pregnancy stress, self-esteem, social support, and antenatal depression. The reliability of each measurement tool was assessed by calculating Cronbach’s α coefficient. Pearson’s correlation coefficients were utilized to examine the relationships between primary variables. PROCESS Macro Model 5 was employed to explore and confirm the mediating and moderating effects within the study model (Supplementary Figure 1). To determine the statistical significance of the mediating effect, bootstrapping was applied, with 5,000 samples re-extracted and a 95% confidence interval (CI) set.

## Results

### General characteristics of participants

The participants’ mean age was 33.52±4.02 years, and their mean gestational age was 23.34±10.56 weeks. Among them, 177 participants (98.3%) were married, and 103 participants (57.2%) were employed, making up the largest group in terms of employment status. In terms of monthly family income, 70 participants (38.9%) earned 5,000,000 Korean won (approximately 40,000 US dollars) or more, which constituted the largest income group. Regarding marital satisfaction, 102 participants (56.7%) reported being “satisfied,” representing approximately half of the sample. In total, 125 participants (69.4%) had planned pregnancies, 123 (69.5%) were primiparous, and 31 (17.2%) had high-risk pregnancies (Supplementary Table 1).

### Levels of antennal depression, pregnancy stress, self-esteem, and social support

The antenatal depression score was 6.51±4.59 points, indicating a very low level. The mean pregnancy stress score was 63.51±14.33 points, suggesting a moderate level. Self-esteem was recorded at 31.06±4.83 points, reflecting a high level, and social support was measured at 41.96±7.73 points, also indicating a high level. The criteria for normality of variables include a skewness of 2 or lower and a kurtosis of 4 or lower; therefore, the primary variables appear to meet these conditions for normality (Table 1).

### Correlations between variables

In this study, antenatal depression was found to have a moderately significant positive correlation with pregnancy stress (r=.52, *p*<.001), a moderately significant negative correlation with self-esteem (r=–.49, *p*<.001), and a slightly significant negative correlation with social support (r=–.24, *p*=.001). Additionally, pregnancy stress exhibited a moderately significant negative correlation with self-esteem (r=–.45, *p*<.001) and a slightly significant negative correlation with social support (r=–.29, *p*<.001). Furthermore, self-esteem showed a moderately significant positive correlation with social support (r=.39, *p*<.001) (Table 2).

### Mediating effect of self-esteem and moderating effect of social support in the impact of pregnancy stress on antenatal depression

**Figure 1.**
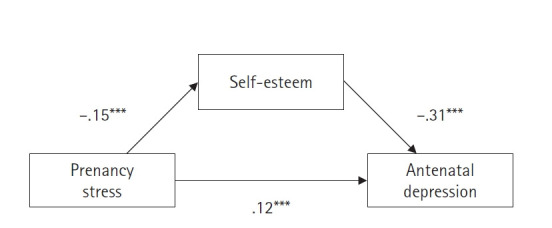
The mediating effect of self-esteem on the relationship between pregnancy stress and antenatal depression.

In this study, Macro Model 5 was utilized to examine the mediating role of self-esteem and the moderating role of social support in the relationship between pregnancy stress and antenatal depression (Table 3). It was determined that pregnancy stress significantly reduced self-esteem (B=–.15, *p*<.001), and lower self-esteem significantly increased antenatal depression (B=–.31, *p*<.001). Additionally, pregnancy stress directly contributed to an increase in antenatal depression (B=.12, *p*<.001). These findings support the significant mediating effect of self-esteem, thereby confirming hypothesis 2 (Figure 1). Moreover, the interaction between pregnancy stress and social support significantly exacerbated antenatal depression (B=–.01, *p*=.036), indicating that the moderating effect of social support is significant and supports hypothesis 3.

Based on the results of the mediating effect analysis, bootstrapping was employed to assess the statistical significance of self-esteem in the relationship between pregnancy stress and postnatal depression. The analysis revealed that pregnancy stress had a total effect of .17 (95% CI, 0.12–0.21), a direct effect of .12 (95% CI, 0.08–0.16), and an indirect effect of .05 (95% CI, 0.02–0.08). The absence of “0” in the 95% CI indicates that the indirect effect of self-esteem is statistically significant (Table 4). These findings suggest that self-esteem serves as a partial mediator in the relationship between pregnancy stress and antenatal depression.

**Figure 2.**
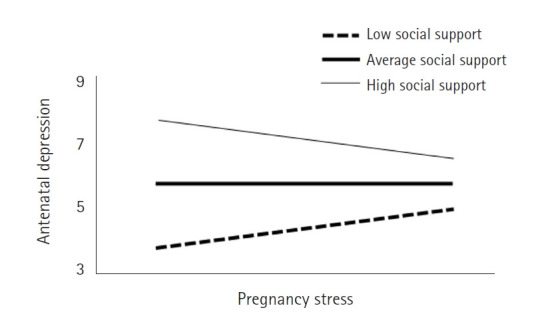
Moderating effect of social support. Social support: low=mean–1 SD, average=mean, high=mean+1 SD.

Moreover, to verify the conditional direct effect, which evaluates how pregnancy stress predicts antenatal depression based on varying levels of social support, we categorized social support into three levels: low (mean–1 SD), average (mean), and high (mean+1 SD). The direct effect of pregnancy stress on antenatal depression was found to be statistically significant across all levels of social support—low, average, and high (Table 3). Additionally, an increase in social support was associated with a decrease in the slope coefficient of the direct effect, moving from B=.15 to .08 (Figure 2).

## Discussion

In this study, self-esteem was examined as a mediating variable and social support as a moderating variable in the relationship between pregnancy stress and antenatal depression. This analysis aimed to explore how pregnancy stress, a known primary cause of antenatal depression, contributes to the condition. The discussion that follows is based on the primary results observed in this study.

First, pregnancy stress has been found to positively correlate with antenatal depression. This finding aligns with previous research [9,32] that identified pregnancy stress as a predictor of antenatal depression, underscoring the importance of managing pregnancy stress to potentially prevent antenatal depression. Women experience various painful physical symptoms, including pelvic pain, morning sickness, and oliguria, along with psychological changes such as fear of childbirth and concerns about baby care and parenting. These factors can contribute to stress [11]. During pregnancy, a woman’s body prepares both her and her fetus for delivery by increasing stress hormone levels, while simultaneously reducing the stress response gradually to prevent excessive stress reactions [33]. If stress persists, hormones like cortisol increase to help manage stressful situations. However, prolonged exposure to high stress levels can elevate the risk of developing depression [34]. Therefore, identifying and managing the sources of stress in pregnant women is a critical strategy for preventing antenatal depression. Nurses should evaluate the stress levels of pregnant women and offer stress management programs that are tailored to each individual’s circumstances and characteristics. Additionally, establishing a social support system that includes family, friends, and a community of pregnant women is essential, as is providing accurate and reliable information about pregnancy, childbirth, and parenting [35].

Second, self-esteem has been identified as a partial mediator in the process by which pregnancy stress leads to antenatal depression. This finding suggests that pregnancy stress not only directly exacerbates symptoms of depression by impacting antenatal depression but also indirectly by reducing self-esteem levels. The study by Jesse et al. [21], conducted with pregnant women, supports this by showing that self-esteem can partially mitigate the effects of stress on the relationship between antenatal stress and antenatal depression, which is consistent with the results of this study. According to DuBois and Flay [36], self-esteem is closely associated with psychological health, acting as a catalyst that enhances resilience. It also serves as a crucial internal resource for pregnant women, interacting with stress to influence antenatal depression [11]. This indicates that self-esteem plays a role in both preventing and managing antenatal depression, as stress during pregnancy can diminish self-esteem, and lowered self-esteem, in turn, increases the likelihood of antenatal depression. Therefore, health centers and obstetric and gynecologic clinics should assess self-esteem in pregnant women, identify those with low self-esteem early, and establish a system for providing intensive counseling. Additionally, given that self-esteem levels vary from one pregnant woman to another, a customized program is essential. Healthcare providers, including obstetricians, gynecologists, nurses, and health center staff, should develop expertise and skills in recognizing the importance of self-esteem and methods to improve it.

Third, social support has been found to moderate the relationship between pregnancy stress and antenatal depression. A study conducted overseas with pregnant Chinese women [22] also demonstrated that social support moderates the relationship between perceived stress and symptoms of antenatal depression. This study indicates that pregnant women with low self-esteem experience higher stress levels and are more likely to exhibit symptoms of antenatal depression, corroborating the findings of this study. Although pregnancy is often a joyful time for women, they undergo significant physical and psychological changes during this period and face increased risks of mental health issues, such as depression. Women with robust social support are less susceptible to antenatal depression, even when experiencing pregnancy stress. Conversely, pregnancy stress is more likely to result in antenatal depression among women with low self-esteem [37]. In essence, social support acts as a protective factor against stress reactivity, helping to mitigate sensitivity to stress [38]. Social support has a positive effect on individuals’ health, independent of stress levels, and reduces the psychological burden by lessening the damage caused by stress [39]. These findings suggest that social support can alleviate the negative impacts of stress and enhance psychological well-being. Therefore, interventions that focus on pregnancy stress and social support are essential for the prevention and treatment of antenatal depression in pregnant women. Since pregnant women are keen to receive a variety of information [20], including details about fetal health, nutritional advice, delivery options, exercises, and customary practices, it is necessary to collect information on their diverse needs related to pregnancy, childbirth, and parenting and to provide them with comprehensive and integrated information. Particularly, since peer support has proven effective in aiding the recovery of women with perinatal depression [37], efforts to strengthen and support a network for information sharing and communication among pregnant women should be intensified.

This study demonstrated the mediating role of self-esteem and the moderating role of social support in the relationship between pregnancy stress and antenatal depression. Specifically, self-esteem partially mediates the impact of pregnancy stress on antenatal depression. Additionally, the influence of pregnancy stress on antenatal depression is contingent upon the level of social support. These findings suggest that interventions aimed at reducing pregnancy stress and enhancing both self-esteem and social support are essential for decreasing antenatal depression among pregnant women.

This study provides meaningful results by suggesting that factors such as social support are effective intervention strategies for the prevention and treatment of antenatal depression. These findings could inform the development of policies aimed at improving the mental health of pregnant women. The study involved a random sample of pregnant women from a hospital, specifically targeting those in the early stages of pregnancy (with an average gestational age of 23 weeks). This timing may have preceded the more pronounced effects of antenatal depression and stress, potentially limiting the applicability of the results to broader populations. Additionally, there is a concern that the stress levels and social support perceived by the participants might not have been accurately captured, given that the assessment tools used were outdated.

The following recommendations can be proposed based on the findings of this study. To generalize the results, further research involving larger medical institutions is required. Additionally, the tools used to measure pregnancy-related stress and social support should be validated and updated to reflect contemporary contexts. It is also essential to develop and evaluate programs aimed at boosting self-esteem and social support to mitigate antenatal depression among pregnant women, based on the insights gained from this study.

## Figures and Tables

**Table 1.** Descriptive statistics of antenatal depression, pregnancy stress, self-esteem, and social support (N=180)

Variable	Possible range	Mean±SD	Item mean±SD	Skewness	Kurtosis
Antenatal depression	0–30	6.51±4.59	0.65±0.45	–.142	–.026
Pregnancy stress	26–130	63.51±14.33	2.44±0.55	.202	.018
Self-esteem	10–40	31.06±4.83	3.11±0.48	.972	.920
Social support	12–60	41.96±7.73	3.50±0.64	–.463	.108

**Table 2.** Correlations among antenatal depression pregnancy stress, self-esteem, and social support (N=180)

Variable	r (*p*)		
	Antenatal depression	Pregnancy stress	Self-esteem
Antenatal depression	1		
Pregnancy stress	.52 (<.001)	1	
Self-esteem	–.49 (<.001)	–.45 (<.001)	1
Social support	–.24 (.001)	–.29 (<.001)	.39 (<.001)

**Table 3.** The mediating effect of self-esteem and the moderating effect of social support on the relationship between pregnancy stress and antenatal depression (N=180)

Variable	B	SE	t	95% CI
Path				
Pregnancy stress → antenatal depression	.12	.02	5.47	0.08 to 0.16
Pregnancy stress → self-esteem	–.15	.02	–6.70	–0.20 to –0.11
Self-esteem → antenatal depression	–.31	.06	–4.77	–0.43 to –0.18
Dependent variable: antenatal depression				
Social support	–.02	.04	–0.42	–0.09 to 0.06
Pregnancy stress × social support	–.01	.00	–2.11	–0.01 to –0.00
Conditional effect				
Social support				
Mean–1 SD	.15	.03	5.44	0.10 to 0.21
Mean	.11	.02	5.34	0.07 to 0.16
Mean+1 SD	.08	.03	2.65	0.02 to 0.13

CI, Confidence interval.

**Table 4.** Bootstrap results for total, direct, and indirect effects

Variable	Effect	Boot SE	Boot 95% CI
Total effect	.17	0.02	0.12–0.21
Direct effect	.12	0.02	0.08–0.16
Indirect effect	.05	0.01	0.02–0.08

CI, Confidence interval.

## Supplementary materials

Further details on supplementary materials are presented online (available at https://doi.org/10.4069/whn.2024.10.18.1).

Supplementary Table 1. General characteristics of the participants (N=180)

Supplementary Figure 1. Hypothesized model.
