# Supplementary material for: Are the effects of stress on antenatal depression mediated by self-esteem and moderated by social support?: a cross-sectional study
Source: Womens Health Nurs. 2024 Dec 30;30(4):299–308. doi: 10.4069/whn.2024.10.18.1 (PMC11700722; doi:10.4069/whn.2024.10.18.1)
Supplement: Supplementary Table 1. — General characteristics of the participants (N=180) [file whn-2024-10-18-1-Supplementary-Table-1.pdf]

Supplementary Table 1. General characteristics of the participants (N=180)

Characteristics	Categories	n (%) or mean ± SD
Age (year)		33.52 ± 4.02
Gestational age (week)		23.34 ± 10.56
Marital status	Married	177 (98.3)
	Not married	3 (1.7)
Employment status	Employed	103 (57.2)
	On leave	28 (15.6)
	Unemployed	49 (27.2)
Family income (Korean won)†	≤ 2 million	3 (1.7)
	2.1–3 million	22 (12.2)
	3.1–4 million	40 (22.2)
	4.1–5 million	45 (25.0)
	≥ 5.1 million	70 (38.9)
Marital satisfaction	Satisfied	102 (56.7)
	Moderate	65 (36.1)
	Unsatisfied	13 (7.2)
Planned pregnancy	Yes	125 (69.4)
	No	54 (30.0)
Parity	Primiparous	125 (69.4)
	Multiparous	55 (30.6)
High-risk pregnancy	Yes	31 (17.2)
	No	149 (82.8)

†1 million Korean won is approximately 800 US dollars
