# Supplementary figures and images for: Are the effects of stress on antenatal depression mediated by self-esteem and moderated by social support?: a cross-sectional study
Source: Womens Health Nurs. 2024 Dec 30;30(4):299–308. doi: 10.4069/whn.2024.10.18.1 (PMC11700722; doi:10.4069/whn.2024.10.18.1)

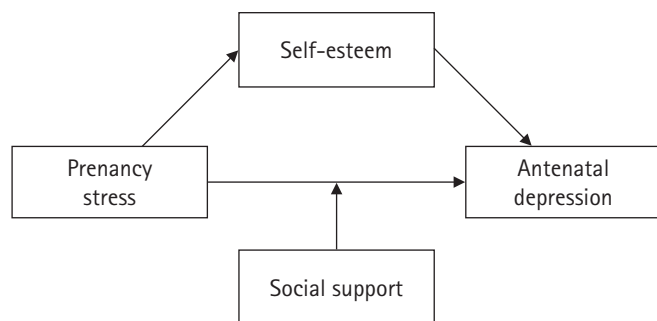

Supplementary Figure 1. Hypothesized model.

Supplement: Supplementary Figure 1. — Hypothesized model. [file whn-2024-10-18-1-Supplementary-Fig-1.pdf]
